# Evolution of the C_4 _phosphoenolpyruvate carboxylase promoter of the C_4 _species *Flaveria trinervia*: the role of the proximal promoter region

**DOI:** 10.1186/1471-2229-8-4

**Published:** 2008-01-21

**Authors:** Sascha Engelmann, Corinna Zogel, Maria Koczor, Ute Schlue, Monika Streubel, Peter Westhoff

**Affiliations:** 1 Institut für Entwicklungs- und Molekularbiologie der Pflanzen, Heinrich-Heine-Universität, Universitätsstr. 1, 40225 Düsseldorf, Germany; 2 Institut für Humangenetik der Universität Duisburg-Essen, Hufelandstr. 55, 45122 Essen, Germany

## Abstract

**Background:**

The key enzymes of photosynthetic carbon assimilation in C_4 _plants have evolved independently several times from C_3 _isoforms that were present in the C_3 _ancestral species. The C_4 _isoform of phosphoenolpyruvate carboxylase (PEPC), the primary CO_2_-fixing enzyme of the C_4 _cycle, is specifically expressed at high levels in mesophyll cells of the leaves of C_4 _species. We are interested in understanding the molecular changes that are responsible for the evolution of this C_4_-characteristic PEPC expression pattern, and we are using the genus *Flaveria *(Asteraceae) as a model system. It is known that *cis*-regulatory sequences for mesophyll-specific expression of the *ppcA1 *gene of *F. trinervia *(C_4_) are located within a distal promoter region (DR).

**Results:**

In this study we focus on the proximal region (PR) of the *ppcA1 *promoter of *F. trinervia *and present an analysis of its function in establishing a C_4_-specific expression pattern. We demonstrate that the PR harbours *cis*-regulatory determinants which account for high levels of PEPC expression in the leaf. Our results further suggest that an intron in the 5' untranslated leader region of the PR is not essential for the control of *ppcA1 *gene expression.

**Conclusion:**

The allocation of *cis*-regulatory elements for enhanced expression levels to the proximal region of the *ppcA1 *promoter provides further insight into the regulation of PEPC expression in C_4 _leaves.

## Background

About 90% of terrestrial plant species, including major crops such as rice, soybean, barley and wheat, assimilate CO_2 _via the C_3 _pathway of photosynthesis. Ribulose-1,5-bisphosphate carboxylase/oxygenase (Rubisco) acts as the primary CO_2_-fixing enzyme of C_3 _photosynthesis, but its ability to use O_2 _as a substrate instead of CO_2 _results in the energy-wasting process of photorespiration. The photosynthetic C_4 _cycle represents an addition to the C_3 _pathway which acts as a pump that accumulates CO_2 _at the site of Rubisco so that the oxygenase activity of the enzyme is inhibited and photorespiration is largely suppressed. C_4 _plants therefore achieve higher photosynthetic capacities and better water- and nitrogen-use efficiencies when compared with C_3 _species [1].

C_4 _photosynthesis is characterized by the coordinated division of labour between two morphologically distinct cell types, the mesophyll and the bundle-sheath cells. The correct functioning of the C_4 _cycle depends upon the strict compartmentalization of the CO_2 _assimilatory enzymes into either mesophyll or bundle-sheath cells [2]. Phosphoenolpyruvate carboxylase (PEPC), which serves as the actual CO_2 _pump of the C_4 _pathway, is specifically expressed in the mesophyll cells of C_4 _leaves. This enzyme is not an unique feature of C_4 _species; other PEPC isoforms with different catalytic and regulatory properties are found in both photosynthetic and non-photosynthetic tissues of all plants where they participate in a variety of metabolic processes, e.g. replenishment of citric acid cycle intermediates and regulation of guard cell movement [3].

The polyphyletic origin of C_4 _photosynthesis suggests that the photosynthetic C_4 _isoforms of PEPC have evolved independently several times from non-photosynthetic C_3 _isozymes [4]. During the evolution of C_4 _PEPC genes from ancestral C_3 _genes, changes in expression strength and organ- and cell-specific expression patterns must have occurred. While C_4 _PEPC genes are highly expressed in the mesophyll cells of the leaf, the C_3 _isoform genes are only moderately transcribed in all plant organs [5-8].

To investigate the molecular evolution of a C_4 _PEPC gene we are using the genus *Flaveria *(Asteraceae) as a model system. This genus includes C_4 _and C_3 _as well as C_3_–C_4 _intermediate species [9,10] and thus provides an excellent system for studying the evolution of the C_4 _photosynthetic pathway [11]. Previous studies on the *ppcA1 *gene of *F. trinervia*, encoding the C_4 _isoform of PEPC, revealed that the strong mesophyll-specific expression is largely regulated at the transcriptional level and that the available 2188 bp (with reference to the AUG start codon of the *ppcA1 *reading frame) of the 5' flanking sequences contain all the essential *cis*-regulatory elements for high and mesophyll-specific expression [12]. Two parts of the *ppcA1 *promoter of *F. trinervia*, a proximal region (PR) up to -570 in combination with a distal region (DR) from -1566 to -2141, are sufficient to direct a high mesophyll-specific expression of a β-glucuronidase (GUS) reporter gene in transgenic *F. bidentis *(C_4_) plants [13]. The orthologous, 2538 bp comprising *ppcA1 *promoter of the C_3 _species *F. pringlei *displays only weak activity in all interior leaf tissues in transgenic *F. bidentis*, but fusion of the C_4_-DR to this C_3 _PEPC promoter leads to a confinement of GUS expression to the mesophyll [13]. Analysis of the C_4_-DR revealed that the 41-bp module MEM1 (mesophyll expression module 1) is responsible for the C_4_-characteristic spatial expression pattern of the *ppcA1 *gene of *F. trinervia*. Furthermore, it was shown that a high level of expression in the mesophyll requires an interaction of the C_4_-DR with the C_4_-PR. This suggests that quantity elements for an elevated expression of the C_4 _PEPC gene are located within the PR of the 5' flanking sequences [13].

Using the yeast one-hybrid system, Windhövel and colleagues [14,15] identified four different proteins which bind to the PR of the *ppcA1 *promoter of *F. trinervia*, but not to the corresponding part of the *ppcA1 *promoter of *F. pringlei*. These proteins (named FtHB1 to FtHB4) belong to the class of zinc finger homeodomain proteins (ZF-HD). Two regions of the C_4_-PR specifically interact with the FtHB proteins *in vitro*: an intron sequence within the 5' untranslated leader region and a DNA fragment that is located upstream of the putative TATA-box. To the latter one, the FtHB proteins showed a much lower binding affinity [14]. Homeobox proteins are known to act as transcriptional regulators of eukaryotic gene expression [16-18], and the fact that the FtHB homeobox proteins interact specifically with the PR of the *ppcA1 *promoter of *F. trinervia *makes them prime candidates for transcription factors that are involved in the establishment of the C_4_-characteristic expression pattern of the C_4 _*ppcA1 *gene.

In this study we have investigated the role of the proximal promoter region of the *ppcA1 *gene of *F. trinvervia *with regard to its high and mesophyll-specific expression by transgenic analyses in the closely related C_4 _species *F. bidentis*. We demonstrate that the proximal promoter region of the *ppcA1 *gene contains *cis*-regulatory elements that determine promoter strength. Furthermore, we show that the deletion of an intron located in the 5' untranslated segment of *ppcA1 *does not alter promoter activity in transgenic *F. bidentis*.

## Results and discussion

### Experimental strategy

**Figure 1:**
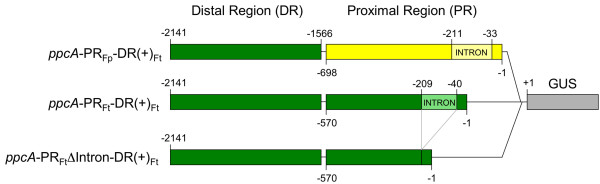
Schematic presentation of the promoter-GUS fusion constructs used for the transformation of *Flaveria bidentis *(C_4_).

We are interested in elucidating the molecular events that are crucial for the evolution of the high and mesophyll-specific expression of the C_4 _phosphoenolpyruvate carboxylase gene (*ppcA1*) of the C_4 _plant *F. trinervia*. In this study we focus on the proximal promoter region (PR) of the *ppcA1 *gene with respect to its function in establishing the C_4_-characteristic expression pattern. We performed a comparative analysis of three different promoter-GUS fusion constructs (Fig. 1) in transgenic *F. bidentis *plants. *F. bidentis *is a close relative to *F. trinervia*, but in contrast to *F. trinervia *this C_4 _species is transformable by *Agrobacterium tumefaciens *mediated gene transfer [19] and was therefore chosen for these experiments.

Construct *ppcA*-PR_Ft_-DR(+)_Ft _served as a reference because it was already known from previous experiments that a combination of the distal (DR) and the proximal (PR) promoter regions was sufficient to direct a high and mesophyll specific expression of a GUS reporter gene in *F. bidentis *[13]. To find out if the PR of the C_4 _*ppcA1 *promoter contains quantity elements conferring high expression in the mesophyll cells we designed construct *ppcA*-PR_Fp_-DR(+)_Ft_. Here, the C_4_-PR was exchanged for its counterpart from the orthologous *ppcA1 *gene of the C_3 _species *F. pringlei*. Deletion of the intron sequences in the 5' untranslated segment of promoter construct *ppcA*-PR_Ft_-DR(+)_Ft _resulted in the formation of construct *ppcA*-PR_Ft_ΔIntron-DR(+)_Ft_. Thereby a putative binding site for the ZF-HD proteins FtHB1 to FtHB4 [14] was removed from the C_4_-PR. Hence, this chimeric promoter-GUS fusion could answer the question whether the intron-located putative binding site of the FtHB proteins is necessary for the establishment of the C_4_-specific *ppcA1 *expression pattern.

### The proximal region of the *ppcA1 *promoter of *F. trinervia *harbours *cis*-regulatory elements for a high level of PEPC expression in the mesophyll

Gowik et al. [13] assumed that the PR of the *ppcA1 *promoter of *F. trinervia *comprises *cis*-regulatory determinants conferring high levels of expression in mesophyll cells of C_4 _leaves. To examine whether the PR actually harbours such quantity elements we analyzed the GUS expression patterns of constructs *ppcA*-PR_Ft_-DR(+)_Ft _and *ppcA*-PR_Fp_-DR(+)_Ft _(Fig. 1) in transgenic *F. bidentis*.

**Figure 2:**
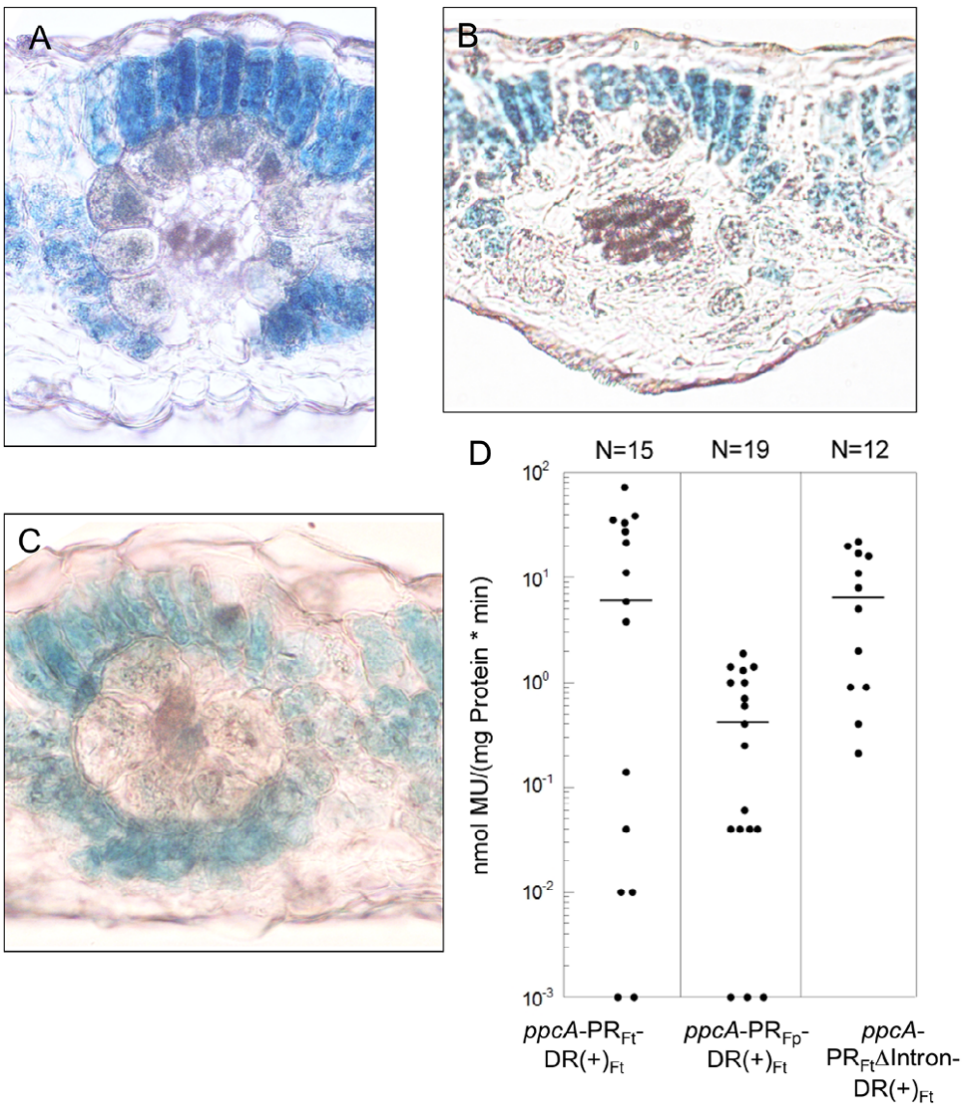
**(A) **to **(C)**: Histochemical localization of GUS activity in leaf sections of transgenic *F. bidentis *plants transformed with constructs *ppcA*-PR_Ft_-DR(+)_Ft_**(A)**, *ppcA*-PR_Fp_-DR(+)_Ft _**(B) **or *ppcA*-PR_Ft_ΔIntron-DR(+)_Ft _**(C)**. Incubation times were 6 h **(A, C) **and 20 h **(B)**. **(D)**: GUS activities in leaves of transgenic *F. bidentis *plants. The numbers of independent transgenic plants tested (N) are indicated at the top of each column. Median values (black lines) of GUS activities are expressed in nanomoles of the reaction product 4-methylumbelliferone (MU) generated per milligram of protein per minute.

In *F. bidentis *plants that had been transformed with promoter construct *ppcA*-PR_Ft-_DR(+)_Ft_, GUS expression was exclusively detected in the mesophyll cells of the leaves (Fig. 2A). This observation shows that the DR and PR of the *ppcA1 *promoter together are sufficient for a high and mesophyll-specific expression of the linked GUS reporter gene and therefore confirms the results obtained by Gowik et al. [13]. Replacement of the C_4_-PR by the corresponding region from the *ppcA1 *promoter of *F. pringlei *(construct *ppcA*-PR_Fp_-DR(+)_Ft_) did not cause any alteration in the cellular GUS expression pattern when compared to *ppcA*-PR_Ft_-DR(+)_Ft_; GUS activity was still restricted to the mesophyll compartment (Fig. 2B). However, both chimeric promoters differed greatly in transcriptional strength. Quantitative GUS assays revealed that promoter activity was decreased by a factor of 15 when the C_4_-PR was substituted for the C_3_-PR (Fig. 2D). This clearly demonstrated that the C_4_-characteristic transcription-enhancing *cis*-regulatory elements must be located within the proximal region of the *ppcA1 *promoter of *F. trinervia*. The low expression level of construct *ppcA*-PR_Fp_-DR(+)_Ft _could be the result of an absence of transcription-enhancing *cis*-regulatory elements in the C_3_-PR, but it might also be caused by problems in the interaction of the C_4_-DR and the C_3_-PR.

### The intron in the C_4_-PR is not required for the establishment of a C_4_-specific expression pattern of the *ppcA1 *gene of *F. trinervia*

The 5' untranslated region of the *ppcA1 *gene of *F. trinervia *contains an intron between positions -209 and -40 (+1 refers to the starting point of translation). Introns are of prominent importance for the molecular evolution of eukaryotic genomes by facilitating the generation of new genes via exon-shuffling and by providing the possibility to create multiple proteins from a single gene via alternative splicing [20-22]. Furthermore, it has been shown that introns can affect many different stages of gene expression, including both transcriptional and post-transcriptional mechanisms [22-24].

Here, we wanted to investigate whether the first intron of the *ppcA1 *gene of *F. trinervia *is essential for establishing the C_4_-characteristic expression pattern. We therefore deleted the intron sequences from the C_4_-PR in construct *ppcA*-PR_Fp_-DR(+)_Ft_, resulting in the formation of construct *ppcA*-PR_Ft_ΔIntron-DR(+)_Ft _(Fig. 1). The histochemical analysis of transgenic *F. bidentis *plants demonstrated that the *ppcA*-PR_Ft_ΔIntron-DR(+)_Ft_promoter was exclusively active in the mesophyll cells of the leaves (Fig. 2C). The quantitative examination of GUS activity (Fig. 2D) also revealed no significant differences between *ppcA*-PR_Ft_ΔIntron-DR(+)_Ft _(6,5 nmol MU/(mg*min)) and *ppcA*-PR_Ft_-DR(+)_Ft _(5,9 nmol MU/(mg*min)). These data suggest that the 5' located intron of *ppcA1 *does not contain any *cis*-regulatory elements that are essential for achieving high mesophyll-specific expression of a reporter gene. Accordingly, the specific binding of the FtHB proteins to this intron that was observed *in vitro *and in yeast one-hybrid experiments [14,15] has no *in planta *relevance concerning the regulation of *ppcA1 *expression in C_4 _leaves. However, our results do not necessarily indicate that the intron is completely dispensable for the regulation of *ppcA1 *gene expression. It is known that C_4 _gene transcription is modulated by various metabolites such as sugar hexoses [25-27], and we cannot exclude that the first intron of the *ppcA1 *gene of *F. trinervia *might be involved in the metabolic control of gene expression.

### Comparison of proximal *ppcA *promoter sequences from different *Flaveria *species

**Figure 3:**
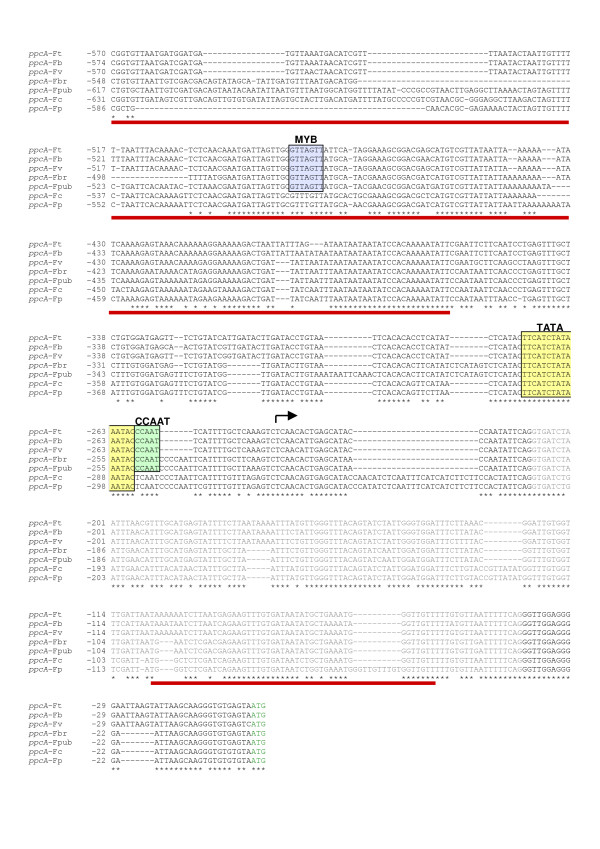
Nucleotide sequence alignment of the proximal regions of *ppcA *promoters from *F. trinervia *(C_4_, *ppcA*-Ft), *F. bidentis *(C_4_, *ppcA*-Fb), *F. vaginata *(C_4_-like, *ppcA*-Fv), *F. brownii *(C_4_-like, *ppcA*-Fbr), *F. pubescens *(C_3_–C_4_, *ppcA*-Fpub), *F. cronquistii *(C_3_, *ppcA*-Fc) and *F. pringlei *(C_3_, *ppcA*-Fp). Identical positions in all *ppcA *sequences are marked by an asterisk. The intron sequences in the 5' untranslated leader regions are marked by grey nucleotides. The start site of the *F. trinervia ppcA *transcript is indicated by an arrow, the TATA-box by a yellow box, the putative MYB-binding site by a blue box, and the CCAAT-sequences by a green box. Fragments of the *F. trinervia ppcA1 *promoter that interact with the FtHB proteins in the yeast one-hybrid system [14, 15] are marked by red bars. The translational ATG start codon is indicated by green nucleotides.

As reported above, *cis*-regulatory elements for leaf-specific enhanced transcription of the *ppcA1 *gene of *F. trinervia *could be allocated to the PR of the 5' flanking sequences, but their exact nature and localization was still unclear. To identify potential *cis*-regulatory enhancing elements, a sequence comparison between the PR of the *ppcA1 *gene of *F. trinervia *and equivalent promoter sequences from other *Flaveria *species was performed (Fig. 3). This approach was chosen because it was already known from northern analyses of *ppcA *transcript levels in different *Flaveria *species that *ppcA *RNA amounts in leaves increase gradually from C_3 _to C_4 _species [28]. This is consistent with the important function of PEPC during C_4 _photosynthesis. The C_4_-like species *F. brownii *and *F. vaginata *exhibited *ppcA *RNA levels that were comparable to those of the C_4 _plants *F. bidentis *and *F. trinervia*, and even in *F. pubescens*, a C_3_–C_4 _intermediate with rather poorly developed C_4_-characteristic traits, *ppcA *transcript accumulation in the leaves was significantly higher than in the C_3 _species *F. cronquistii *and *F. pringlei *[28].

Searching for known plant *cis*-regulatory DNA elements in the PLACE database [29] resulted in the identification of two distinct sequence motifs which might be involved in the regulation of *ppcA *expression levels (Fig. 3). Both of them, a putative MYB transcription factor binding site (GTTAGTT, [30]) and a CCAAT box [31], are present in all examined C_3_–C_4_, C_4_-like and C_4 _species, but are missing in the two C_3 _species (Fig. 3). Thus, these sequences are prime candidates for transcription-enhancing *cis*-regulatory elements. CCAAT boxes are common sequences that are found in the 5' untranslated regions of many eukaryotic genes [32]. They are able to regulate the initiation of transcription by an interaction of CCAAT-binding transcription factors with the basal transcription initiation complex [33]. There is no unifying expression pattern for plant genes containing putative CCAAT promoter elements, indicating that they may play a complex role in regulating plant gene transcription [32]. MYB proteins, on the other hand, comprise one of the largest families of transcription factors in plants, with almost 200 different MYB genes present in the Arabidopsis genome [34-36]. To test the physiological importance of the putative MYB and CCAAT binding sites (that are located within the PR of the *ppcA1 *promoter of *F. trinervia*) it will be crucial to inactivate these sequences in construct *ppcA*-PR_Ft_ΔIntron-DR(+)_Ft _by site-directed mutagenesis and to investigate whether this results in a decrease of reporter gene expression in the leaves of transgenic *F. bidentis *plants.

When searching for quantity elements in the PR of the *ppcA1 *promoter of *F. trinervia*, one should always keep in mind that high levels of reporter gene expression in the leaf mesophyll require the synergistic action of the distal and proximal promoter regions. The C_4_-PR alone exhibits very low transcriptional activity in all interior leaf cell types of transgenic *F. bidentis *[37], indicating that the *cis*-regulatory elements for enhanced expression are only functional when the C_4_-PR is combined with the cognate C_4_-DR. One may speculate that a strong expression of the *ppcA1 *gene in the mesophyll cells of *F. trinervia *depends on the interaction of *trans*-acting factors which bind to *cis*-regulatory elements within the PR with other transcription factors that are recruited to C_4_-specific *cis*-regulatory determinants in the DR. In the future, further dissection of the C_4_-PR of *F. trinervia *and expression analyses of additional DR-PR combinations from *ppcA *promoters of different *Flaveria *species in transgenic *F. bidentis *will be useful for uncovering the control of *ppcA *expression levels in C_4 _leaves.

## Conclusion

In this study, we have demonstrated that the proximal region (-570 to -1) of the *ppcA1 *promoter of *F. trinervia *(C_4_) harbours *cis*-regulatory elements conferring high expression levels in leaf mesophyll cells of transgenic *F. bidentis *(C_4_). It was further demonstrated that the deletion of an intron in the 5' untranslated leader region does not affect the C_4_-specific *ppcA1 *expression pattern and strength, indicating that the previously isolated zinc finger-homeobox transcription factors that specifically interact with this intron *in vitro *are not involved in regulating *ppcA1 *expression levels. Sequence comparisons resulted in the identification of potential *cis*-regulatory elements in the proximal part of the *ppcA1 *promoter that might play a role in controlling *ppcA1 *expression quantity. Genetic manipulation of these sequences and subsequent analyses in transgenic *F. bidentis *will clarify whether they are able to direct high *ppcA1 *expression levels in C_4 _leaves.

## Methods

### Construction of chimeric promoters

DNA manipulations and cloning were performed according to Sambrook and Russell [38]. The construction of the promoter-GUS fusion *ppcA*-PR_Ft_-DR(+)_Ft _has been described in detail [13]. Plasmids *ppcA*-S-Fp[39] and *ppcA*-PR_Ft_-DR(+)_Ft _served as the basis for the production of *ppcA*-PR_Fp_-DR(+)_Ft_. The distal region (-2141 to -1566) of the *ppcA1 *promoter of *F. trinervia *was excised from *ppcA*-PR_Ft_-DR(+)_Ft _by digestion with *Xba*I. Insertion of this promoter fragment into *Xba*I-cut *ppcA*-S-Fp resulted in the generation of construct *ppcA*-PR_Fp_-DR(+)_Ft_.

For the production of construct *ppcA*-PR_Ft_ΔIntron-DR(+)_Ft _a part of the *ppcA1 *promoter from *F. trinervia *(-570 to -209) was amplified by PCR with primers S-Ft-F (5'-TGCTCTAGACCGGTGTTAATGATGG-3') and S-Ft-R (5'-CTGAATATTGGGTATG-CTCAG-3'). Plasmid *ppcA*-PR_Ft_-DR(+)_Ft _was used as the template for this PCR reaction. The amplified promoter fragment was cut with *Xba*I. The outermost 3' region of the *ppcA1 *promoter (-39 to -1) was generated by annealing the two oligonucleotides S-Ft-3'-1 (5'-GGTTGGAGGGGAATTAAGTATTAAGCAAGGGTGTGAGTAC-3') and S-Ft-3'-2 (5'-CCGGGTACTCACACACCCTTGCTTAATACTTAATTCCCCTCCAACC-3'). Thereby a *Xma*I-compatible 5' overhang was created next to position -1. The *ppcA*-S-Ft promoter plasmid [39] was digested with *Xba*I and *Xma*I and the released *ppcA1 *promoter fragment was removed by agarose gel electrophoresis. The *Xba*I/*Xma*I-cut *ppcA*-S-Ft plasmid was ligated with the two *ppcA1 *promoter fragments (-570 to -209/-39 to -1) and the resulting plasmid was named *ppcA*-PR_Ft_ΔIntron. The distal region of the *ppcA1 *promoter of *F. trinervia *(-2141 to -1566) was removed from of *ppcA*-PR_Ft_-DR(+)_Ft _by incubation with *Xba*I and inserted into *Xba*I-cut *ppcA*-PR_Ft_ΔIntron. The resulting plasmid was designated *ppcA*-PR_Ft_ΔIntron -DR(+)_Ft_.

### Plant transformation

In all transformation experiments the *Agrobacterium tumefaciens *strain AGL1 was used [40]. The promoter-GUS constructs were introduced into AGL1 by electroporation. The transformation of *Flaveria bidentis *was performed as described by Chitty et al. [19]. The integration of the transgenes into the genome of regenerated *F. bidentis *plants was proved by PCR analyses.

### Measurement of GUS activity and histochemical analysis

*F. bidentis *plants used for GUS analysis were 40 to 50 cm tall and before flower initiation. Fluorometrical quantification of GUS activity in the leaves was performed according to Jefferson et al. [41] and Kosugi et al. [42]. For histochemical analysis of GUS activity the leaves were cut manually with a razorblade and the sections were transferred to incubation buffer (100 mM Na_2_HPO_4_, pH 7.5, 10 mM EDTA, 50 mM K_4 _[Fe(CN)_6_], 50 mM K_3 _[Fe(CN)_6_], 0.1% (v/v) Triton X-100, 2 mM 5-bromo-4-chloro-3-indolyl-β-D-glucuronid acid). After brief vacuum infiltration the sections were incubated at 37°C for 6 to 20 hrs. After incubation chlorophyll was removed from the tissue by treatment with 70% ethanol.

### Computer analyses

DNA sequence analyses were performed with MacMolly Tetra [43]. The sequence alignments were created with the program DIALIGN 2.2.1 [44]. Sequence data mentioned in this article can be found in GenBank under accession numbers X64143 (*F. trinervia ppcA1*), X64144 (*F. pringlei ppcA1*), AY297090 (*F. vaginata ppcA1*), AY297089 (*F. cronquistii ppcA1*), AY297087 (*F. bidentis ppcA1*), EF522173 (*F. brownii ppcA1*) and EF522174 (*F. pubescens ppcA1*).

## Authors' contributions

SE carried out the histochemical and quantitative GUS assays, the cloning of construct *ppcA*-PR_Ft_ΔIntron-DR(+)_Ft_, the sequence alignments and wrote the manuscript. CZ produced construct *ppcA*-PR_Fp_-DR(+)_Ft_. MK, US and MS performed the transformation of *F. bidentis*. PW coordinated the design of this study and participated in drafting the manuscript. All authors read and approved the final manuscript.
